# Speech, Gait, and Vestibular Function in Cerebellar Ataxia with Neuropathy and Vestibular Areflexia Syndrome

**DOI:** 10.3390/brainsci13101467

**Published:** 2023-10-17

**Authors:** Giulia Di Rauso, Andrea Castellucci, Francesco Cavallieri, Andrea Tozzi, Valentina Fioravanti, Edoardo Monfrini, Annalisa Gessani, Jessica Rossi, Isabella Campanini, Andrea Merlo, Dario Ronchi, Manuela Napoli, Rosario Pascarella, Sara Grisanti, Giuseppe Ferrulli, Rossella Sabadini, Alessio Di Fonzo, Angelo Ghidini, Franco Valzania

**Affiliations:** 1 Department of Biomedical, Metabolic and Neural Science, University of Modena and Reggio Emilia, 41125 Modena, Italy; giuliadirauso3@gmail.com; 2 Neurology, Neuroscience Head Neck Department, Azienda Ospedaliero-Universitaria di Modena, 41126 Modena, Italy; gessani.annalisa@aou.mo.it; 3 Neurology Unit, Neuromotor & Rehabilitation Department, Azienda USL-IRCCS di Reggio Emilia, 42123 Reggio Emilia, Italy; valentina.fioravanti@ausl.re.it (V.F.); jessica.rossi@ausl.re.it (J.R.); rossella.sabadini@ausl.re.it (R.S.); franco.valzania@ausl.re.it (F.V.); 4 Otolaryngology Unit, Azienda USL-IRCCS di Reggio Emilia, 42123 Reggio Emilia, Italy; andrea.castellucci@ausl.re.it (A.C.); angelo.ghidini@ausl.re.it (A.G.); 5 Otorhinolaryngology-Head and Neck Surgery Department, University Hospital of Modena, 41125 Modena, Italy; andreatozzi29@gmail.com (A.T.); dottorgiuseppeferrulli@gmail.com (G.F.); 6 Neurology Unit, Foundation IRCCS Ca’ Granda Ospedale Maggiore Policlinico, 20122 Milan, Italy; edoardo.monfrini@unimi.it (E.M.); dario.ronchi@unimi.it (D.R.); alessio.difonzo@policlinico.mi.it (A.D.F.); 7 Dino Ferrari Center, Neuroscience Section, Department of Pathophysiology and Transplantation, University of Milan, 20122 Milan, Italy; 8 Clinical and Experimental Medicine PhD Program, University of Modena and Reggio Emilia, 41125 Reggio Emilia, Italy; grisanti.sara@gmail.com; 9 LAM-Motion Analysis Laboratory, Neuromotor and Rehabilitation Department, Azienda USL-IRCCS di Reggio Emilia, 42123 Reggio Emilia, Italy; isabella.campanini@ausl.re.it (I.C.); andrea.merlo@ausl.re.it (A.M.); 10 Neuroradiology Unit, Azienda USL-IRCCS di Reggio Emilia, 42123 Reggio Emilia, Italy; manuela.napoli@ausl.re.it (M.N.); rosario.pascarella@ausl.re.it (R.P.)

**Keywords:** ataxia, balance, CANVAS, dysarthria, gait, RFC1, speech, vestibular areflexia

## Abstract

(1) Background: Cerebellar ataxia with neuropathy and vestibular areflexia syndrome (CANVAS) is characterized by late-onset cerebellar ataxia, bilateral vestibulopathy, and sensory neuronopathy mostly due to biallelic RFC1 expansion. (2) Objectives: The aim of this case series is to describe vestibular, gait, and speech alterations in CANVAS via a systematic approach. (3) Methods: All patients (n = 5) underwent a standardized clinical–instrumental examination, including the perceptual and acoustic analysis of speech, instrumental gait, and balance analysis (posturographic data were acquired using a force plate [Kistler, Winterthur, Switzerland] while 3D gait analysis, inclusive of surface electromyography, was acquired using a motion capture system [SMART DX, BTS Bioengineering, Milan, Italy], a wireless electromyograph [FreeEMG, BTS Bioengineering, Milan, Italy]), and vestibular assessment with video-oculography. (4) Results: Five patients were included in the analysis: three females (patients A, B, C) and two males (patients D and E) with a mean age at evaluation of 62 years (SD ± 15.16, range 36–74). The mean age of symptoms’ onset was 55.6 years (SD ± 15.04, range 30–68), and patients were clinically and instrumentally evaluated with a mean disease duration of 6.4 years (SD ± 0.54, range 6–7). Video-Frenzel examination documented spontaneous downbeat nystagmus enhanced on bilateral gaze in all patients, except for one presenting with slight downbeat nystagmus in the supine position. All patients exhibited different degrees of symmetrically reduced VOR gain for allsix semicircular canals on the video-head impulse test and an unexpectedly normal (“false negative”) VOR suppression, consistent with combined cerebellar dysfunction and bilateral vestibular loss. Posturographic indices were outside their age-matched normative ranges in all patients, while 3D gait analysis highlighted a reduction in ankle dorsiflexion (limited forward rotation of the tibia over the stance foot during the stance phase of gait and fatigue of the dorsiflexor muscles) and variable out-of-phase activity of plantar flexors during the swing phase. Finally, perceptual-acoustic evaluation of speech showed ataxic dysarthria in three patients. Dysdiadochokinesis, rhythm instability, and irregularity were observed in the oral diadochokinesis task. (5) Conclusions: CANVAS is a recently discovered syndrome that is gaining more and more relevance within late-onset ataxias. In this paper, we aimed to contribute to a detailed description of its phenotype.

## 1. Introduction

Cerebellar ataxia with neuropathy and vestibular areflexia syndrome (CANVAS) is a slowly progressive form of hereditary late-onset ataxia characterized by bilateral vestibulopathy, cerebellar dysfunction, and somatic sensory perception deficit [1,2,3,4,5,6]. This syndrome has emerged progressively over the last 30 years [1,2,3,4,5,6], and recently, the biallelic pentanucleotide expansion in intron 2 of the replication factor C subunit 1 (RFC1) was identified as the main cause of CANVAS [1,2,3,4,5,6].

The main neurological symptoms at onset are postural imbalance, typically worsened by darkness with falls and/or dizziness; patients also report sensory symptoms or oscillopsia [1]. A chronic cough is also considered part of the clinical spectrum [1,2,3,4,5,6] and may occur before the appearance of neurological symptoms [1,2,3,4,5,6]. The main clinical triad is composed of cerebellar ataxia, neuronopathy, or vestibular areflexia [1,2,3,4,5,6].

The absence or severe reduction in the vestibulo-ocular reflex (VOR) is the clinical sign of bilateral vestibular dysfunction [1,2,3,4,5,6], while the contemporary deficit of vestibular and cerebellar ocular motor stabilizing functions causes a deficit of visually enhanced VOR (VVOR) [1,2,3,4,5,6]. Video-oculography (VOG) can detect and measure V-VOR and VOR deficits, particularly in the early phase when they could be clinically subtle [7].

Moreover, non-length dependent multimodality sensory deficit due to sensory neuropathy is a typical clinical feature, and it could manifest with the impairment of vibration and tactile sensations, kinesthetic state deficit, limb ataxia, and, more rarely, pain/temperature perception dysfunction [7]. Electroneuromyography typically shows sensory axonal neuropathy. Neuropathology studies revealed peripheral and vestibular ganglionopathy [1].

A single previous study described the posturographic data of four patients who met the diagnostic criteria for definitive CANVAS (without, however, genetic test confirmation). The study highlighted severe alteration in the sensory organization test (SOT), while observing normal limits of stability (LOS) [8]. Cerebellar dysfunction is typically characterized by dysarthria, truncal and appendicular ataxia, and oculomotor cerebellar signs, including gaze-evoked and downbeat nystagmus and saccadic dysmetria [1].

MRI is the standard technique for brain evaluation in patients with possible CANVAS. These patients usually do not display pathognomonic MRI abnormalities, but a reproducible pattern of atrophy involving cerebellar structures, especially the cerebellar vermis, was described [1,7,9,10].

However, to date, no studies have instrumentally assessed speech and gait alterations in genetically confirmed CANVAS, and it is unclear if its characteristic cerebellar neurodegeneration may lead to a different degree of the involvement of gait and speech functions.

Based on these premises, the objective of this study is to describe, via a systematic clinical–instrumental approach, vestibular dysfunction, speech, and gait alterations in five patients with genetically confirmed CANVAS in order to contribute to a detailed description of the phenotype, which could facilitate its recognition and early diagnosis.

## 2. Materials and Methods

This case series included five consecutive patients with a genetic diagnosis of CANVAS in follow-up at the Neurology Unit of AUSL-IRCCS of Reggio Emilia. A brain MRI was performed. Moreover, all patients underwent a systematic instrumental assessment in order to evaluate vestibular function, speech, and gait parameters.

### 2.1. Auditory, Vestibular and Oculomotor Assessment

#### 2.1.1. Hearing Function

Pure-tone audiometry was performed over the frequency range of 125 to 8000 Hz and 250 to 4000 Hz for air conduction and bone conduction, respectively, once the normal status of tympanic membranes was ascertained in micro-otoscopic examination. The pure-tone average for both air-conducted sounds was calculated across 500 to 4000 kHz and compared to age-related normality ranges. As for impedance audiometry, standard tympanometry with a 226-Hz probe tone and ipsi/contralateral acoustic reflexes were administered to all patients.

#### 2.1.2. Assessment of Spontaneous and Induced Nystagmus

**Figure 1:**
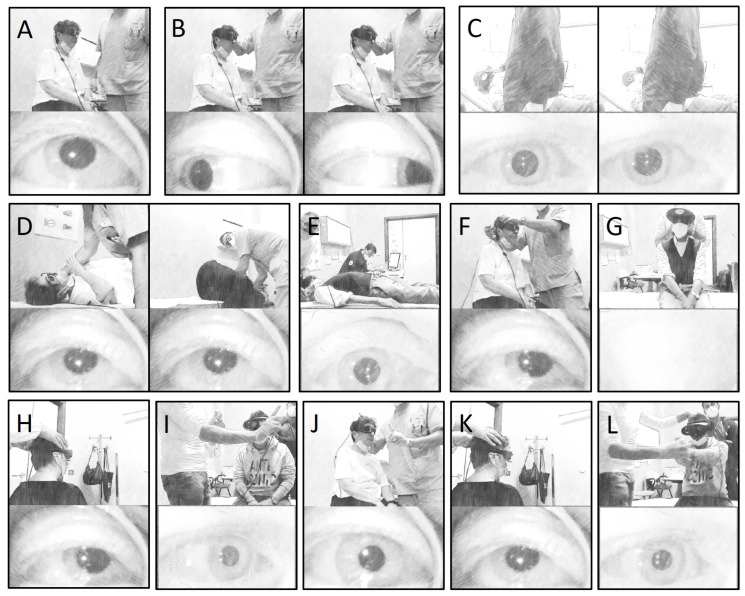
Vestibular and oculomotor evaluation via an infra-red video-Frenzel system with a field camera (upper panel) and an eye camera (lower panel). It included the assessment of spontaneous nystagmus (**A**), gaze-evoked/rebound nystagmus (**B**), positional nystagmus evoked by the supine head-roll test (**C**), Dix–Hallpike/Semont positionings on both sides (**D**) and/or straight head-hanging position (**E**), skull vibration-induced nystagmus (**F**), and head shaking nystagmus (**G**). Video-head impulse testing (**H**). Evaluation of saccadic (**I**) and smooth pursuit movements (**J**). Assessment of visually enhanced VOR (**K**) and VOR suppression tests (**L**).

Eye movements were recorded either with a monocular or with a binocular infra-red video-Frenzel system. Horizontal, vertical, and torsional components of nystagmus were qualitatively assessed. Horizontal (right/leftbeating), vertical (upbeating/downbeating) directions of nystagmus, and torsional (right/left) components were described from the patient’s point of view. The video-Frenzel examination included the assessment of spontaneous nystagmus, gaze-evoked/rebound nystagmus, and positional nystagmus evoked by the supine head-roll test, Dix–Hallpike/Semont positionings on both sides and/or straight head-hanging position. For the assessment of spontaneous nystagmus, the patient was instructed to look straight ahead from the upright position (Figure 1A). The patient was then asked to look first on one side and then to look back into the center, and then to do the same on the other side to search for gaze-evoked/rebound nystagmus (Figure 1B). Then, the patient was asked to perform the supine head-roll test lying supine with the head first on one side and then to the other (Figure 1C). Similarly, they were brought from the upright to the supine position with the head hyperextended and turned 45° first on one side and then on the other, according to Dix–Hallpike/Semont positionings (Figure 1D), and finally along the sagittal plane, according to the straight head-hanging position (Figure 1E). Spontaneous and positional nystagmus were classified according to the predominant features: absent, present, upbeat, or downbeat. Skull vibration and head-shaking tests were conducted with the patient upright. Skull vibration-induced nystagmus was elicited by applying a handheld 100-Hz vibrator (VVIB 100 Hz Synapsys, Marseille, France) to both mastoids for at least 5–10 s (Figure 1F). Skull vibration-induced nystagmus was considered reliable only if vibrations in both mastoids resulted in the same oculomotor pattern. Then, 30 cycles of passive head rotations were imparted at a rate of 1–2 Hz, and the post head-shaking nystagmus was evaluated in the 30 s following the test (Figure 1G). Both skull vibration-induced nystagmus and post-head-shaking nystagmus were classified as absent, horizontal (right/leftbeating), and vertical (up/downbeating) [11].

#### 2.1.3. Video-Head Impulse Test

The video-head impulse test was performed to evaluate the vestibulo-ocular reflex (VOR) gain for each semicircular canal using an ICS video-oculographic system (GN Otometrics, Taastrup, Denmark). Passive, unpredictable 150°–250°/s and 3000°–5000°/s^2^ head impulses were delivered manually on the plane of the horizontal and vertical SCs while the patient was asked to keep looking at an earth-fixed target, according to the standard protocol [12] (Figure 1H). At least 15 stimuli were delivered for stimulating each SC and averaged to obtain the corresponding mean VOR gain. VOR gain values < 0.8 for horizontal SC and <0.7 for anterior and posterior SC with corrective saccades (overt and/or covert) were considered pathological [12].

#### 2.1.4. Oculomotor Testing

Saccadic eye movements were quantitively measured using a random saccade test with a monocular ICS video-oculographic system (GN Otometrics, Denmark). The stimulus used for the randomized saccade was a horizontal visual target presented randomly at different angles in the range of 0–20° (to the left and right) and at a constant frequency (0.8 Hz) for a duration of 60 s (Figure 1I). Measurement parameters included saccadic peak velocity (degrees/s), accuracy/precision (%), and latency (ms). Conversely, smooth pursuit movements were quantitatively assessed on bedside examination and classified as normal or abnormal (saccadic). The patient was instructed to focus on a target moving horizontally in a pendular pattern (Figure 1I).

#### 2.1.5. Visually Enhanced VOR and VOR Suppression Tests

Both visually enhanced VOR (VVOR) and VOR suppression (VORS) tests were qualitatively assessed using a monocular ICS video-oculographic system (GN Otometrics, Denmark). As for the VVOR test, the patient’s head was slowly rotated horizontally from side to side while the patient stared at an earth-fixed target from the sitting position (Figure 1K). To evaluate the ability of the vestibolocerebellum to cancel the VOR, the patient’s head was rotated on the horizontal plane in a pendular pattern, and the patient was instructed to focus on a head-fixed target. Alternatively, while seated, the patient was first asked to extend his/her arms directly in front of the body, clasping the hands together with thumbs pointed upward, then he/she was asked to visually fixate on his/her own thumbs and maintain that fixation. The patient’s entire body was then rotated “en bloc” from side to side (Figure 1L). Both VVOR and VORS tests were classified as normal or abnormal (saccadic).

### 2.2. Speech Assessment

A perceptual and acoustic analysis of speech was conducted to assess the patterns and degree of dysarthria. A speech evaluation based on analysis of sustained phonation (vowels/i/and/a/), diadochokinesis (ddk) with repetition of alternated syllables (pa-ta-ka), spontaneous speech (the patients were asked to tell a fairy tale) and reading of a passage was performed. Word intelligibility was also calculated by the percentage of words correctly transcribed by the examiner (GDR, who was blinded to patients’ recording and was not familiar with the patient’s speech patterns) among a set of 25 recorded words. Single-word intelligibility was selected due to its advantage of eliminating a number of other variables that can affect intelligibility, such as sentence-level syntactic and prosodic variables. Furthermore, the use of single words to assess intelligibility is a much less difficult task for dysarthric participants than sentence-level productions. Perceptual and acoustic data were collected using the open-source Praat software^®^ version 6.3.18 [13] and the free beta version of Dysarthria Analyzer software. Some of the variables included in the analysis were maximum phonation time (MPT) [s], intensity (dB), a fraction of locally unvoiced frames, and a number of voice breaks (these parameters were evaluated during sustained phonation tasks); speech rate (syllables/second) calculated during counting tasks; rhythm instability; net speech rate (NSR); rhythm acceleration; diadochokinetic (ddk) standard deviation of power (stdPWR); DDK rate; and vowel duration [14,15,16]. Evaluations were made at a silent voice conversation intensity (<50 dB of background noise), and each evaluation was recorded using a microphone that was kept 20 cm from the patient’s lips. The severity of dysarthria was perceptually determined by a speech–language pathologist (AG) and was categorized on a coarse scale ranging from none, mild, moderate to severe (1: severe; 2: moderate; 3: mild; 4: none) [17,18]. The Dysarthria subtype was perceptually characterized based on the Darley, Aronson, and Brown dysarthria subtypes classification [19,20].

### 2.3. Instrumental Gait and Balance Analysis

Posturographic data were acquired using a force plate (Kistler, Winterthur, Switzerland). The protocol [21] consisted of a sequence of five 30 s tasks, as follows: standing with eyes open on a firm surface; standing with eyes closed on a firm surface; standing with eyes open on a firm surface while performing a cognitive task referred to as a dual task; and standing with eyes open on a compliant surface and standing with eyes closed on a compliant surface. The compliant surface was obtained by placing a viscoelastic gel pillow (Elastil II, Laboratoires Escarius, La Courneuve, France) over the force plate. Three repetitions per task were acquired to ensure data consistency. Three-dimensional gait analysis (3DGA), inclusive of surface electromyography, was acquired using a motion capture system (SMART DX, BTS Bioengineering, Milan, Italy) and a wireless electromyograph (FreeEMG, BTS Bioengineering, Milan, Italy). Retroreflective markers were placed over the skin, according to the Conventional Protocol [22,23,24,25]. Electrodes were placed over the target muscles on the minimal crosstalk areas [22,23,24,25]. Five walking trials at self-selected speeds were recorded. Instrumented Timed Up&Go (iTUG) was acquired using a wearable inertial unit (gSensor, BTS Bioengineering, Milan, Italy) worn at the S1 level. Three repetitions were acquired both in normal conditions and while performing a cognitive task [26,27].

## 3. Results

### 3.1. Demographic Data

Five consecutive patients with a diagnosis of CANVAS were included in the analysis: three females (patients A, B, C) and two males (patients D and E) with a mean age at evaluation of 62 years (SD ± 15.16, range 36–74). The mean age of symptoms’ onset was 55.6 years (SD ± 15.04, range 30–68), and patients were clinically and instrumentally evaluated with a mean disease duration of 6.4 years (SD ± 0.54, range 6–7).

### 3.2. Clinical Presentation

All patients complained of progressive unsteadiness of gait with postural imbalance, and three of them complained of oscillopsia during fast head movements with neither acute vertigo spells nor auditory symptoms. All patients were walking independently at the time of evaluation without the need to use assistive devices and were independent in the activities of daily living (ADLs) and instrumental activities of daily living (IADLs). Two patients (patients A and C) reported paresthesia in the extremities, while the remaining three (patients B, D, and E) presented hypoesthesia in the same districts. In addition, all patients underwent a nerve conduction study, which confirmed the presence of moderate sensory axonal polyneuronopathy. Nobody presented symptoms suggestive of dysautonomia or dysphagia. Finally, all patients have a history of unexplained coughs from youth. Table 1 shows the main clinical features presented by the five patients, while detailed clinical characteristics of the patients are summarized in Supplementary Materials.

### 3.3. Genetic Testing

The RFC1 gene expansion was assessed by flanking PCR, showing no detectable wild-type alleles. Using repeat-primed polymerase chain reaction, the pathological AAGGG pentanucleotide expansion could be detected in both alleles of the RFC1 gene, while non-pathogenic AAAGG or AAAAG expansions were excluded, confirming the genetic diagnosis of CANVAS.

### 3.4. Vestibular Function Testing with Video-Oculography (VOG)

**Figure 2:**
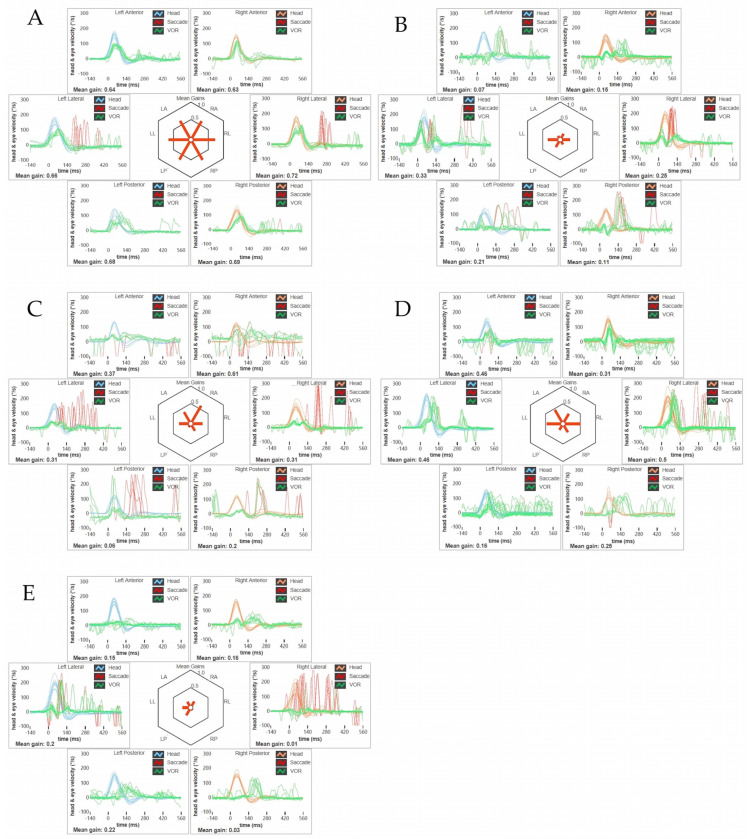
Video-Head Impulse Test. (**A**) Patient A: vHIT showing a slight symmetrical reduction in all the SC on both sides with corrective saccades in all planes. Blue lines represent head impulses exciting left canals; orange lines correspond to impulses for right canals; green lines represent eye movements induced by the activation of VOR following each impulse; and red lines correspond to corrective saccades. Mean value of VOR gain (eye velocity/head velocity) is reported for each canal. The hexagonal plot in the center of the figure summarizes mean VOR gains for each canal; impaired gains are shown in red. (**B**) Patient B: vHIT highlighting a symmetrical severe functional impairment of all the SC with corrective saccades. (**C**) Patient C: vHIT showing a severe bilateral reduction in all the SC with corrective saccades, with the exception of the ASC, which exhibits only slightly reduced VOR gain values (0.61). (**D**) Patient D: vHIT detecting a symmetrical impairment of the VOR gain values of all the SC with corrective saccades. (**E**) Patient E: vHIT depicting a dramatic symmetrical reduction in the VOR gain values of all the SC with corrective saccades. *LA*: *left anterior*, *LL*: *left lateral*, *LP*: *left posterior*, *RA*: *right anterior*, *RL*: *right lateral*, *RP*: *right posterior*, *ASC*: *anterior semicircular canal*, *SC*: *semicircular canals*, *vHIT*: *video-Head Impulse Test*, *VOR*: *vestibulo-ocular reflex*.

**Figure 3:**
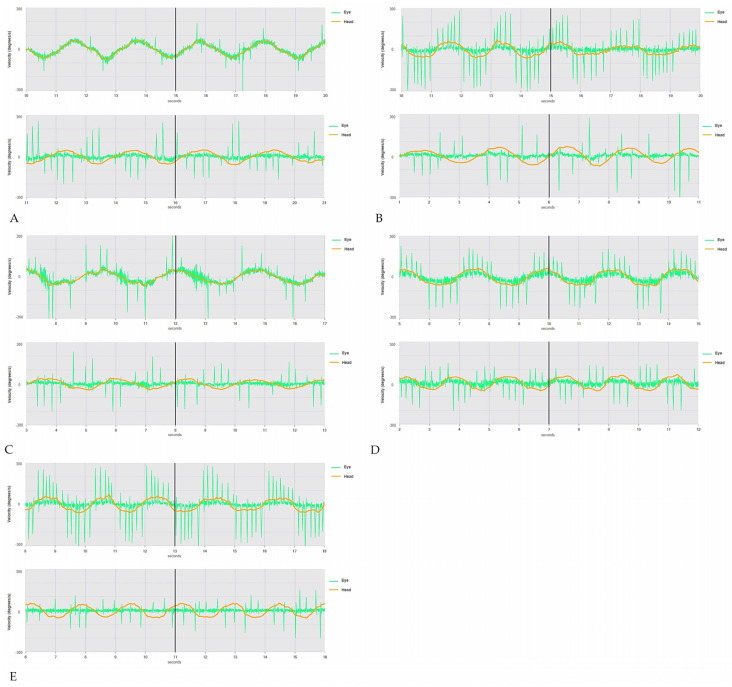
VVOR and VORS tests. (**A**) Patient A: VVOR (above) and VORS (below) tests showing almost normal eye movements. Right eye movements are in green, and head movements are in orange. (**B**) Patient B: Abnormal VVOR test with high-amplitude saccades (above) and almost normal VORS test (below). (**C**) Patient C: VVOR test showing low-amplitude saccadic eye movements (above) and near-normal VORS test (below). (**D**) Patient D: Abnormal VVOR test with high-amplitude saccades (above) and near-normal VOR cancellation (below). (**E**) Patient E: Abnormal VVOR test with high-amplitude saccades (above) and near-normal VORS test (below). *VOR*: *vestibulo-ocular reflex*. *VORS*: *VOR suppression*, *VVOR*: *visually enhanced VOR*.

All patients exhibited either normal hearing function or symmetrical high-frequency impairment consistent with their age, and impedance audiometry was within normal limits in all cases. Video-Frenzel examination documented spontaneous downbeat nystagmus (DBN), slightly reducing its amplitude in supine positionings in four patients. The amplitude of spontaneous DBN increased on both lateral gazes where an outward horizontal component was superimposed, resulting in a laterally/downward-directed gaze-evoked nystagmus (side-pocket nystagmus) [28]. One patient (patient A) presented with only slight positional DBN in supine positionings. The head-shaking test modified neither the direction nor the intensity of the spontaneous DBN in any case, whereas a right beating nystagmus could be elicited by skull vibrations in one case (patient C). All patients exhibited different degrees of VOR gain reduction for all six semicircular canals (SCs) on the video-head impulse test (vHIT). While only one patient (patient A) developed a slight symmetrical impairment for all SCs on both sides, a severe symmetrical VOR-hypofunction could be detected in the other subjects (patients B, C, D, and E). In patient C, the right anterior SC exhibited only slightly reduced VOR gain values, leading to an asymmetrical functional impairment between the two vestibular organs (Figure 2). While patient A exhibited near-normal oculomotor tests, the other four patients presented saccadic pursuit. Sporadic eye movements consistent with ocular flutter could be detected in only one case (patient B). Three subjects (patients B, D, and E) exhibited saccadic dysmetria (both undershoots and overshoots in two cases; only undershoots in one case). The peak velocity of horizontal saccades was slightly low in two subjects (patients B and D), while saccade latency was slightly increased in only one case (patient C) (Supplementary Material—Figure S1). In three patients, clear high-amplitude saccades replacing smooth eye movements could be detected at the VVOR test (patients B, D, and E), while in the other two cases, VVOR was only mildly impaired (patients A and C). All patients exhibited a normal/near-normal VOR suppression (VORS) test (Figure 3). Video-oculography and videos of bedside evaluation of HIT in the five patients are included in Supplementary Materials.

### 3.5. Neuroimaging Findings

All patients underwent 1.5 T brain MRI, and different degrees of cerebellar atrophy were found in all of them. As described in the literature [1,9,10], infratentorial atrophy involves vermian structures, especially in the superior and dorsal aspect (lobules VI, VIIa, and VIIb) and both cerebellar lobes with predilection of crus I and with the secondary widening of the superior, posterior, and horizontal fissures. No supratentorial specific anomalies or brain atrophy were detected. Two subjects (A and C) also presented small multiple scattered areas of T2/FLAIR white matter hyperintensity that were consistent with chronic small vessel ischemia. Four patients (A, B, C, and D) performed spinal MRI (1.5 T); one subject (patient D) showed T2 hyperintensity on the dorsal and cervical spinal cord, and one (patient B) had mild volume reduction in the spinal cord. Images of the most significant neuroradiological findings of the patients included in this study are included in the Supplementary Materials Figure S2.

### 3.6. Perceptual and Acoustic Analysis of Speech

The assessment of speech in the patients examined revealed mixed results. Indeed, two out of five patients (patients B and C) did not show any speech alterations, while the remaining three patients (patients A, D, and E) showed a mild to moderate ataxic dysarthric pattern. In addition, one of these three patients (patient D) presented a mixed pattern of spastic-ataxic dysarthria, which was in line with clinical–instrumental findings (i.e., upper motor neuron signs and white matter lesions on brain and spinal cord MRI). The acoustic analysis of speech documented in three out of five patients (patients A, D, and E) the presence of acoustic alterations in oral diadochokinesis (rhythm irregularity, vowel duration, standard deviation of power [stdPWR]) in the rhythm of spontaneous speech (rhythm instability), in reading (slow speech; syllables/second). Interestingly, no acoustic alterations were found in the sustained phonation, while only one patient (patient E) presented a harsh voice, a perceptual finding typically seen in ataxic dysarthria. The perceptual analysis of spontaneous monologue also showed a slightly scanned speech in three patients (patients A, D, and E); the voice emission was sometimes explosive with aggregations and distortions of consonants and consonant clusters. All these alterations were compatible with ataxic dysarthria. Table 2 shows the speech profile presented by the five patients in detail.

### 3.7. Instrumental Gait and Balance Analysis

Posturographic indices (mean velocity of the center of pressure and area) were outside their age-matched normative ranges. These further worsened on a compliant surface or with closed eyes. No patient could perform the test on a compliant surface with closed eyes. The dual-task condition led to indices similar to those of the baseline assessment for all subjects. Completing the Instrumented Timed Up&Go (iTUG) test required 11–17 s among patients without worsening consequent to the dual-task condition, except for patient D. Gait temporal and spatial parameters (e.g., velocity, cadence, step length, etc.) were in their normative ranges for all patients but patient D, who walked at 0.53 m/s with a reduction in the duration of the single limb support and an increase in the duration of the double supports. This indicates a deficit in maintaining dynamic balance. In all patients, during the stance phase of gait, 3D gait analysis (3DGA) highlighted a reduction in ankle dorsiflexion, i.e., a limited forward rotation of the tibia over the stance foot. In addition, during the swing phase, the fatiguability of dorsiflexor muscles and variable out-of-phase activity of plantar flexors were also found.

## 4. Discussion

### 4.1. Oculomotor and Vestibular Findings

Due to the slow progressive course of the bilateral vestibular failure in CANVAS, patients’ history typically lacks acute vertigo spells. Patients usually experience oscillopsia, unsteadiness, and dynamic imbalance aggravated by head movements as a result of the compound of both vestibular and cerebellar dysfunction [7]. In particular, while bilateral vestibular areflexia results in dynamic oscillopsia due to a deficient VOR for all the SCs [29], cerebellar impairment involving floccular and parafloccular areas can result in spontaneous and/or positional DBN accounting for static oscillopsia [11,28,30]. Either spontaneous or positional DBN was found in all the patients of our series. Overall signs of cerebellar impairment comprise a wide range of abnormal eye movements in addition to DBN, including gaze-evoked nystagmus (GEN), saccadic pursuit, dysmetric saccades, periodic alternating nystagmus, central positional nystagmus and impaired VORS [11,28,30].

Sometimes, they might coexist in the same patient according to the different patterns of cerebellar involvement. The so-called “side-pocket nystagmus” represents the compound of DBN and GEN [28], and it was detected in four patients of our series. Similarly, the association of different degrees of abnormalities involving saccadic movements, including hypometric and hypermetric saccades as detected in three patients, likely reflects different degrees of functional impairment of the cerebellar vermis [28,30]. Similarly, the detection of saccadic intrusions consistent with ocular flutter in a patient of our series likely reflects a lesion involving the oculomotor vermis [28].

Nevertheless, since CANVAS represents a model of combined peripheral and central disease, the characteristic oculomotor sign is an abnormal VVOR, which reflects a combined deficit of three compensatory oculomotor reflexes, including VOR, optokinetic reflex, and smooth pursuit [28]. Only three patients in our series (patients B, D, and E) exhibited high-amplitude saccades, replacing smooth movements at the VVOR test. The remaining two patients (patients A and C) exhibited poor clinical signs of abnormal VVOR test; hence, they might be included in the so-called “CANVAS in evolution” population [31]. On the other hand, it is also possible to ascertain a functional impairment of both peripheral and central pathways via a detailed vestibular and oculomotor assessment. The VOR can be measured by testing SCs function in different frequency ranges: caloric irrigations measure low-frequency afferents, the rotatory chair assesses mild-frequency VOR, while the HIT was demonstrated to selectively evaluate the activity of the SCs in the high-frequency domain. While the first two techniques can only assess the horizontal SC afferents, the recent introduction of the vHIT has enabled clinicians to easily measure the VOR gain for all six SCs [12]. Similarly, central oculomotor pathways can be easily assessed by testing saccadic movements, smooth pursuits, and VOR cancellation, which shares the same neural network as the smooth pursuit system. In cases of pure cerebellar dysfunction involving the vestibolocerebellum, besides saccadic dysmetria, both smooth pursuits and VORS should be abnormal, revealing saccadic eye movements [28].

Nevertheless, in the case of CANVAS, the coexistence of bilateral vestibular hypofunction of peripheral origin results in an unexpectedly normal VOR cancellation [7,31]. In fact, similar to other degenerative diseases affecting both central and peripheral vestibular pathways, a “falsely negative” cancellation test might be misleading in patients with absent VOR function, as there is no need to suppress any VOR by the vestibolocerebellum. Therefore, an unexpected dissociation between smooth pursuit deficits and normal VOR cancellation represents a key point in the diagnosis of CANVAS.

In our series, most patients (patients B, C, D, and E) presented with saccadic pursuit, whereas all of them exhibited unexpectedly normal VORS. Nevertheless, as some other conditions might result in selective impairment of smooth pursuit with preserved VORS despite no signs of VOR hypofunction, the vHIT should always be given prior to assessing and interpreting VOR cancellation test data [32]. Moreover, vestibular hypofunction related to CANVAS usually affects all peripheral vestibular receptors and afferents in a symmetrical pattern, so that the clinical and instrumental tests unmasking vestibular asymmetries, such as positional tests, head shakings, and skull vibrations, are usually uneventful. Accordingly, no signs of vestibular asymmetry could be found in four patients (patients A, B, D, and E). Conversely, rightbeating components of nystagmus elicited by skull vibrations in one patient of our series (patient C) likely reflect a functional asymmetry between the vestibular end-organs, in particular between the right anterior SC (only slightly affected) and the functionally paired contralateral posterior SC (severely affected). In fact, it might be hypothesized that the greater impairment of left posterior SC resulted in unopposed mild excitation of the right anterior SC, leading to an enhancement of spontaneous DBN with right beating components after skull vibrations [33].

### 4.2. Balance and Gait Findings

The instrumental assessment allowed us to quantify an impaired balance ability in all patients, which further worsened when visual or somatosensory inputs were altered. This worsening reflects the pathophysiological basis of CANVAS, which affects three key components of the balance system (i.e., peripheral sensory inputs from the proprioceptive and vestibular systems; cerebellar control function) [34]. On the contrary, the impaired balance ability did not worsen when an additional cognitive task was required, except for one patient. This is not surprising considering that CANVAS is not usually associated with cognitive alterations, which may alter the ability to divide attention between cognitive and motor activity during the dual-task condition leading to a worsening of balance performance [35]. The only patient who worsened during the dual task (patient D) presented the contemporary presence of white matter lesions [36], which may have affected the execution of the dual-task condition, well-reported findings, for example, in Multiple Sclerosis patients [37].

Interestingly, the same pattern was also confirmed in the iTUG test, in which only patient D showed a worsening during the dual-task condition. In addition, in all patients, 3DGA highlighted an initial impairment in ankle kinematics that was not appreciated in the clinical observation, along with an altered timing of plantar flexor muscles, which was similar between the patients.

### 4.3. Speech Findings

While consistent results were obtained from balance and gait analysis, with the presence of alterations in balance and gait abilities in all patients, perceptual and acoustic assessment of speech showed heterogeneous results. Indeed, only two out of five patients did not show any speech alterations, while on the contrary, the remaining three patients showed a mild to moderate ataxic dysarthric pattern.

This finding suggests that patients with CANVAS may present a heterogenous involvement of speech as opposed to stance and gait alterations. Functional MRI studies have localized speech articulation mainly in the medial parts of lobule VI bilaterally and crus 1 [38,39] with no localization within the vermian lobules. This differs from stance and gait alterations, which are mainly localized in the medial and intermediate cerebellum [38], whose atrophy and neurodegeneration represent the mainstay of the pathologically definite CANVAS [9].

The dysarthric patterns found in our cases well reflected the neuroimaging and clinical findings. Indeed, while patient D presented mild spastic-ataxic dysarthria, which may be linked to the contemporary presence of white matter lesions that may justify the spastic component of dysarthria, patients A and E presented pure ataxic dysarthria, which reflected the selective involvement of the intermediate cerebellum. This is not trivial, particularly if considering the differential diagnosis of CANVAS [9]. Indeed, as an example, dysarthria associated with Friedreich’s Ataxia (FRDA) is not always purely ataxic in nature but may rather involve a mix of ataxic/spastic/flaccid components [40].

On the contrary, the motor speech impairments associated with SCA3 and SCA6 are generally classified as ataxic dysarthria with a differential involvement of specific speech parameters [41]. Indeed, the regularity of DDK was reported to be specifically impaired in SCA3, whereas impairments of other speech parameters, i.e., rate and modulation, were strongly affected in SCA6. In our CANVAS patients, both the regularity of oral diadochokinesis and the rate of reading tasks were altered. Obviously, future studies with a larger sample are needed to assess the presence of possible differences in specific speech parameters between CANVAS and its differential diagnoses.

## 5. Conclusions

We report a case series of five patients affected by CANVAS with distinctive clinical–instrumental findings, including ataxic dysarthria, ataxic gait, and bilateral vestibulopathy with oculomotor abnormalities consistent with cerebellar dysfunction. CANVAS is a recently discovered syndrome that is gaining more and more relevance within late-onset ataxias. With this paper, we aimed to contribute to a detailed description of its phenotype. In fact, in our opinion, it would be worth describing in detail the CANVAS phenotype in order to better know its pathophysiology and to facilitate its recognition and early diagnosis.

## Figures and Tables

**Table 1:** Main clinical features in patients.

Symptoms	Patient A	Patient B	Patient C	Patient D	Patient E
Cough	^•^	^•^	^•^	^•^	^•^
Gait unsteadiness	^•^	^•^	^•^	^•^	^•^
Postural imbalance	^•^	^•^	^•^	^•^	^•^
Limb ataxia	^•^	^•^	°	^•^	^•^
Sensory symptoms	^•^	^•^	^•^	^•^	^•^
Dysarthria	^•^	^•^	^•^	^•^	^•^
Dysautonomia	°	°	°	°	°
Oscillopsia	^•^	°	^•^	°	^•^

^•^ Presence of symptom; ° Absence of symptom.

**Table 2:** Speech characteristics in CANVAS patients.

	Patient A	Patient B	Patient C	Patient D	**Patient E**
**Motricity and preliminary observations**	no alterations	no alterations	no alterations	no alterations	no alterations
**Spontaneous speech**	mild scanned speech, articulatory distortions of consonants, and consonant clusters. Acoustic analysis showed rhythm instability (z 2.05).	no alterations	no alterations	slightly reduced intelligibility that requires attention from the listener, slightly nasal and pressed voice, sometimes explosive emission, aggregations, and distortions of consonants and consonant clusters. Acoustic analysis showed rhythm instability (z 2.78).	slightly reduced intelligibility that requires attention from the listener, harsh voice, scanned speech, aggregations and distortions of consonants and consonant clusters. Acoustic analysis showed abnormally high rhythm acceleration (z 5.79).
**Reading passage**	reduced NSR (z −2.06)	no alterations	no alterations	reduced NSR (z −1.92)	reduced NSR (z −2.61)
**Oral diadochokinesis**	the rapid production of the single syllable/pa/shows irregular rhythm (z score 2.05), which is also evident in the alternating production of syllables with different points of articulation. The ddk stdPWR was abnormally high (z 3.72) and was associated with rhythm instability (z 1.87).	no alterations	no alterations	the rapid production of the single syllable/pa/shows irregular rhythm (z score 2.78), which is also evident in the alternating production of syllables with different points of articulation with abnormally high ddk stdPWR (z 2.08), rhythm instability (z 1.81) and vowel duration (z 2.08).	the rapid production of the single syllable/pa/shows irregular rhythm (z score 5.39), which is also evident in the alternating production of syllables with different points of articulation with abnormally high rhythm instability (z 3.27), vowel duration (z 16.20) and ddk stdPWR (z 9.45). On the contrary, the DDK rate was abnormally low (z −4.59).
**Single words speech intelligibility**	100%	100%	100%	100%	88%
**Sustained phonation letters/i/and/a/**	no alterations	no alterations	no alterations	no alterations	harsh voice
**Perceptual pattern of dysarthria**	mild ataxic dysarthria	no dysarthric speech	no dysarthric speech	mild spastic-ataxic dysarthria	moderate ataxic dysarthria

## Data Availability

The data presented in this study are available on request from the corresponding author. The data are not publicly available due to ethical restrictions.

## Supplementary Materials

The following supporting information can be downloaded a https://www.mdpi.com/article/10.3390/brainsci13101467/s1. Video S1. Video-Frenzel examination of patient #A; Video S2. Video-Frenzel examination of patient #B; Video S3. Video-Frenzel examination of patient #C; Video S4. Video-Frenzel examination of patient #D; Video S5. Video-Frenzel examination of patient #E; Figure S1: Analysis of saccadic movements; Figure S2: Main neuroradiological findings; Table S1: Detailed description of patients’ clinical characteristics.

## References

[1] Dupré M., Hermann R., Froment Tilikete C. (2021). Update on Cerebellar Ataxia with Neuropathy and Bilateral Vestibular Areflexia Syndrome (CANVAS). Cerebellum.

[2] Migliaccio A.A. (2004). Cerebellar Ataxia with Bilateral Vestibulopathy: Description of a Syndrome and Its Characteristic Clinical Sign. Brain.

[3] Szmulewicz D.J., Waterston J.A., Halmagyi G.M., Mossman S., Chancellor A.M., McLean C.A., Storey E. (2011). Sensory Neuropathy as Part of the Cerebellar Ataxia Neuropathy Vestibular Areflexia Syndrome. Neurology.

[4] Wu T.Y., Taylor J.M., Kilfoyle D.H., Smith A.D., McGuinness B.J., Simpson M.P., Walker E.B., Bergin P.S., Cleland J.C., Hutchinson D.O. (2014). Autonomic Dysfunction Is a Major Feature of Cerebellar Ataxia, Neuropathy, Vestibular Areflexia “CANVAS” Syndrome. Brain.

[5] Petersen J.A., Wichmann W.W., Weber K.P. (2013). The Pivotal Sign of CANVAS. Neurology.

[6] Cortese A., Simone R., Sullivan R., Vandrovcova J., Tariq H., Yau W.Y., Humphrey J., Jaunmuktane Z., Sivakumar P., Polke J. (2019). Biallelic Expansion of an Intronic Repeat in RFC1 Is a Common Cause of Late-Onset Ataxia. Nat. Genet..

[7] Szmulewicz D.J., Waterston J.A., MacDougall H.G., Mossman S., Chancellor A.M., McLean C.A., Merchant S., Patrikios P., Halmagyi G.M., Storey E. (2011). Cerebellar Ataxia, Neuropathy, Vestibular Areflexia Syndrome (CANVAS): A Review of the Clinical Features and Video-Oculographic Diagnosis. Ann. N. Y. Acad. Sci..

[8] de la Roca-Morales A.M.M., Andreo-Marroig J.F., Santos-Pérez S., Soto-Varela A. (2018). Instability in Patients with CANVAS: Can Computerized Dynamic Posturography Help in Diagnosis?. J. Int. Adv. Otol..

[9] Szmulewicz D.J., Roberts L., McLean C.A., MacDougall H.G., Halmagyi G.M., Storey E. (2016). Proposed Diagnostic Criteria for Cerebellar Ataxia with Neuropathy and Vestibular Areflexia Syndrome (CANVAS). Neurol. Clin. Pract..

[10] Szmulewicz D.J., McLean C.A., Rodriguez M.L., Chancellor A.M., Mossman S., Lamont D., Roberts L., Storey E., Halmagyi G.M. (2014). Dorsal Root Ganglionopathy Is Responsible for the Sensory Impairment in CANVAS. Neurology.

[11] Brandt T. (2003). Vertigo: Its Multisensory Syndrome.

[12] Halmagyi G.M., Chen L., MacDougall H.G., Weber K.P., McGarvie L.A., Curthoys I.S. (2017). The Video Head Impulse Test. Front. Neurol..

[13] Boersma P., Weenink D. Praat: Doing Phonetics by Computer [Computer Program]. Version 5.3.51. 2013. [Online]. http://www.praat.org/.

[14] Hlavnička J., Růžičková H., Tykalová T., Novotny M., Rusz J. Dysarthria Analyzer. Beta Version. 2022; ([Computer Program]). http://www.dysan.cz/.

[15] Cavallieri F., Budriesi C., Gessani A., Contardi S., Fioravanti V., Menozzi E., Pinto S., Moro E., Valzania F., Antonelli F. (2020). Dopaminergic Treatment Effects on Dysarthric Speech: Acoustic Analysis in a Cohort of Patients with Advanced Parkinson’s Disease. Front. Neurol..

[16] Cavallieri F., Di Rauso G., Gessani A., Budriesi C., Fioravanti V., Contardi S., Menozzi E., Pinto S., Moro E., Antonelli F. (2023). A Study on the Correlations between Acoustic Speech Variables and Bradykinesia in Advanced Parkinson’s Disease. Front. Neurol..

[17] Yorkston K.M., Miller R.M., Strand E.A. (2004). Management of Speech and Swallowing in Degenerative Diseases.

[18] Hartelius L., Elmberg M., Holm R., Lövberg A.-S., Nikolaidis S. (2008). Living with Dysarthria: Evaluation of a Self-Report Questionnaire. Folia Phoniatr. Logop..

[19] Darley F.L., Aronson A.E., Brown J.R. (1969). Differential Diagnostic Patterns of Dysarthria. J. Speech Hear. Res..

[20] Rowe H.P., Gutz S.E., Maffei M.F., Tomanek K., Green J.R. (2022). Characterizing Dysarthria Diversity for Automatic Speech Recognition: A Tutorial from the Clinical Perspective. Front. Comput. Sci..

[21] Merlo A., Zemp D., Zanda E., Rocchi S., Meroni F., Tettamanti M., Recchia A., Lucca U., Quadri P. (2012). Postural Stability and History of Falls in Cognitively Able Older Adults: The Canton Ticino Study. Gait Posture.

[22] Merlo A., Campanini I. (2019). Impact of Instrumental Analysis of Stiff Knee Gait on Treatment Appropriateness and Associated Costs in Stroke Patients. Gait Posture.

[23] Campanini I., Merlo A., Disselhorst-Klug C., Mesin L., Muceli S., Merletti R. (2022). Fundamental Concepts of Bipolar and High-Density Surface EMG Understanding and Teaching for Clinical, Occupational, and Sport Applications: Origin, Detection, and Main Errors. Sensors.

[24] Campanini I., Cosma M., Manca M., Merlo A. (2020). Added Value of Dynamic EMG in the Assessment of the Equinus and the Equinovarus Foot Deviation in Stroke Patients and Barriers Limiting Its Usage. Front. Neurol..

[25] Campanini I., Merlo A., Damiano B. (2013). A Method to Differentiate the Causes of Stiff-Knee Gait in Stroke Patients. Gait Posture.

[26] Cavallieri F., Campanini I., Gessani A., Budriesi C., Fioravanti V., Di Rauso G., Feletti A., Damiano B., Scaltriti S., Guagnano N. (2023). Long-Term Effects of Bilateral Subthalamic Nucleus Deep Brain Stimulation on Gait Disorders in Parkinson’s Disease: A Clinical-Instrumental Study. J. Neurol..

[27] Cavallieri F., Gessani A., Merlo A., Campanini I., Budriesi C., Fioravanti V., Di Rauso G., Feletti A., Damiano B., Scaltriti S. (2023). Interplay between Speech and Gait Variables in Parkinson’s Disease Patients Treated with Subthalamic Nucleus Deep Brain Stimulation: A Long-Term Instrumental Assessment. Eur. J. Neurol..

[28] Leigh R.J., Zee D.S. (2015). The Neurology of Eye Movements.

[29] Strupp M., Kim J.-S., Murofushi T., Straumann D., Jen J.C., Rosengren S.M., Della Santina C.C., Kingma H. (2017). Bilateral Vestibulopathy: Diagnostic Criteria Consensus Document of the Classification Committee of the Bárány Society. J. Vestib. Res..

[30] Zwergal A., Feil K., Schniepp R., Strupp M. (2020). Cerebellar Dizziness and Vertigo: Etiologies, Diagnostic Assessment, and Treatment. Semin. Neurol..

[31] Szmulewicz D.J., McLean C.A., MacDougall H.G., Roberts L., Storey E., Halmagyi G.M. (2014). CANVAS an Update: Clinical Presentation, Investigation and Management. J. Vestib. Res..

[32] Takeichi N., Fukushima K., Sasaki H., Yabe I., Tashiro K., Inuyama Y. (2000). Dissociation of Smooth Pursuit and Vestibulo-Ocular Reflex Cancellation in SCA-6. Neurology.

[33] Dumas G., Curthoys I.S., Lion A., Perrin P., Schmerber S. (2017). The Skull Vibration-Induced Nystagmus Test of Vestibular Function-A Review. Front. Neurol..

[34] Bronstein A.M., Pavlou M. (2013). Balance. Handb. Clin. Neurol..

[35] Li K.Z.H., Bherer L., Mirelman A., Maidan I., Hausdorff J.M. (2018). Cognitive Involvement in Balance, Gait and Dual-Tasking in Aging: A Focused Review from a Neuroscience of Aging Perspective. Front. Neurol..

[36] Azzimonti M., Fazio R., Giordano A., Tagliapietra M., Ferrarini M., Rocca M.A., Fabrizi G.M., Filippi M., Colombo B. (2022). Association between Inflammatory Central Nervous System Lesions and Cerebellar Ataxia, Neuropathy and Vestibular Areflexia Syndrome (CANVAS): A Case Series. J. Neurol..

[37] Beste C., Mückschel M., Paucke M., Ziemssen T. (2018). Dual-Tasking in Multiple Sclerosis—Implications for a Cognitive Screening Instrument. Front. Hum. Neurosci..

[38] Timmann D., Brandauer B., Hermsdörfer J., Ilg W., Konczak J., Gerwig M., Gizewski E.R., Schoch B. (2008). Lesion-Symptom Mapping of the Human Cerebellum. Cerebellum.

[39] Argyropoulos G.P.D., Watkins K.E., Belton-Pagnamenta E., Liégeois F., Saleem K.S., Mishkin M., Vargha-Khadem F. (2019). Neocerebellar Crus I Abnormalities Associated with a Speech and Language Disorder Due to a Mutation in FOXP2. Cerebellum.

[40] Folker J., Murdoch B., Cahill L., Delatycki M., Corben L., Vogel A. (2010). Dysarthria in Friedreich’s Ataxia: A Perceptual Analysis. Folia Phoniatr. Logop..

[41] Brendel B., Synofzik M., Ackermann H., Lindig T., Schölderle T., Schöls L., Ziegler W. (2015). Comparing Speech Characteristics in Spinocerebellar Ataxias Type 3 and Type 6 with Friedreich Ataxia. J. Neurol..

