# Supplementary material for: Speech, Gait, and Vestibular Function in Cerebellar Ataxia with Neuropathy and Vestibular Areflexia Syndrome
Source: Brain Sci. 2023 Oct 17;13(10):1467. doi: 10.3390/brainsci13101467 (PMC10605709; doi:10.3390/brainsci13101467)
Supplement: Supplementary file 1 [file brainsci-13-01467-s001.zip › Supplementary materials.pdf]

Supplementary materials

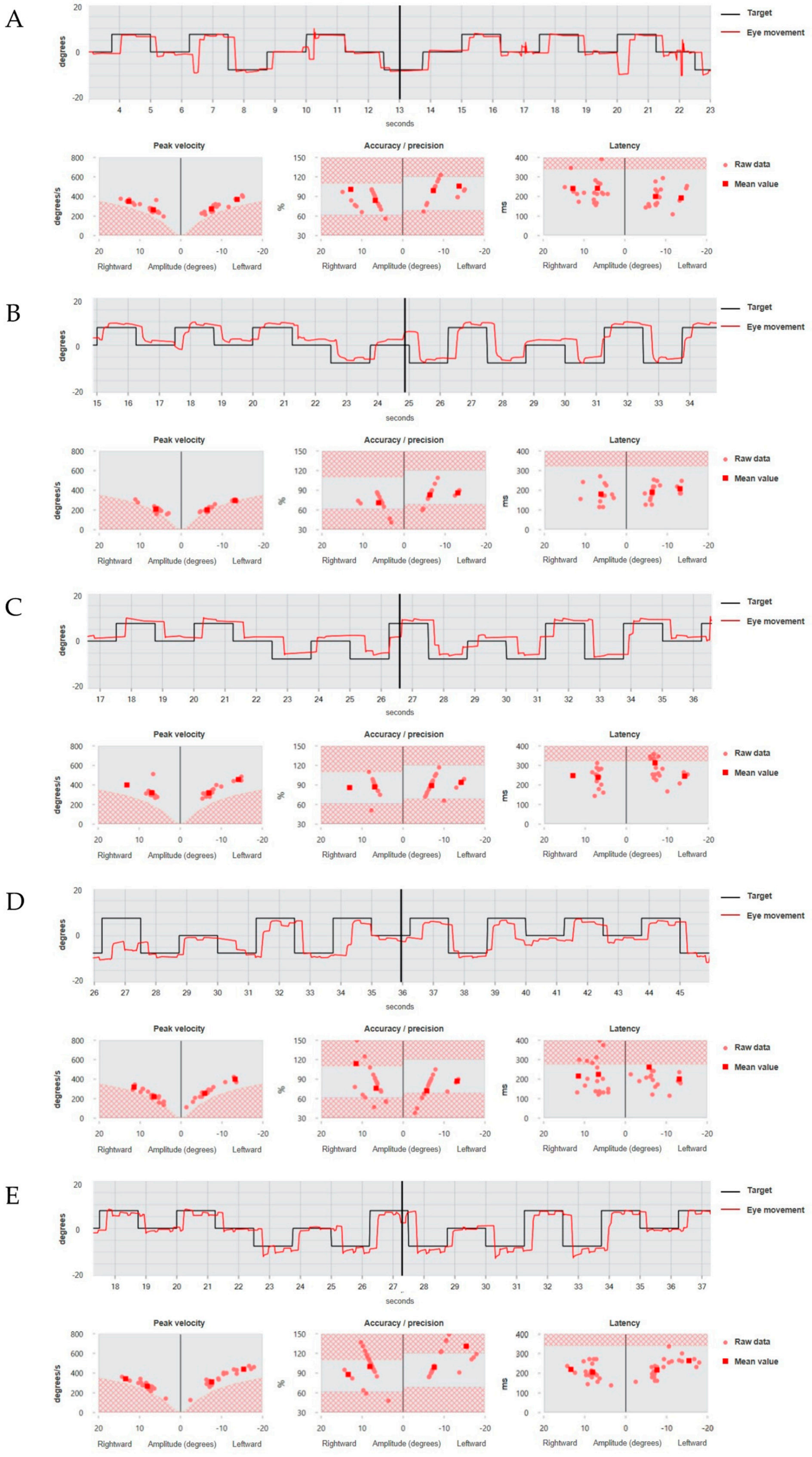

Figure S1. Analysis of saccadic movements

(A) Patient A: Right eye position tracking (above) and corresponding analyses of saccadic movements (below) with areas in red squared background corresponding to age-related abnormality ranges. The analyses show mean values for peak velocity, accuracy/precision and latency within normality ranges. **(B)** Patient B: Analysis of saccades showing slightly low mean values of peak velocity, hypometric saccades and normal latency. **(C)** Patient C: Analysis of saccadic movements showing overall normal values of peak velocity and accuracy/precision, while highlighting slightly increased values for latency. **(D)** Patient D: Saccade analysis shows slightly abnormal (low) mean values of peak velocity, both undershoots and overshoots, and slightly increased mean value for latency. **(E)** Patient E: Saccadic movements present with normal mean values of peak velocity and latency, and saccadic dysmetria.

SC: semicircular canals, vHIT: video-Head Impulse Test, VOR: vestibulo-ocular reflex. VORS: VOR-suppression, VVOR: visually-enhanced VOR.

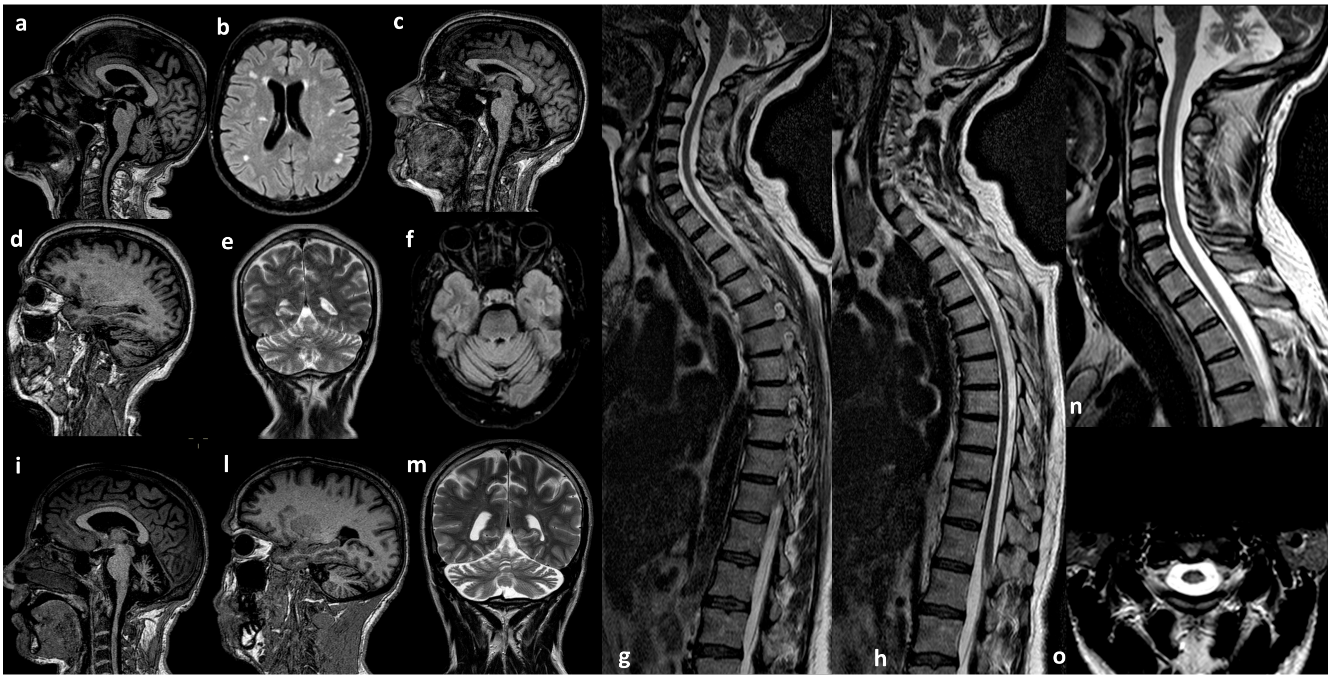

Figure S2. Main neuroradiological findings. Patient A: T1 sagittal midsagittal image (a): mild vermian atrophy in middle-superior segments. FLAIR axial image (b): multiple areas of signal hyperintensity scattered in the deep and peripherical WM. Patient B: T1 sagittal brain images (c: midsagittal; d: parasagittal), Coronal T2 brain image (e), Axial FLAIR brain image (f), T2 sagittal cervical spinal image (g) and T2 sagittal dorsal spinal image (h): (c-f) Vermian atrophy, especially in the superior and dorsal aspect (lobules VI, VIIa, and VIIb). (d-e-f) cerebellar lobar atrophy predominantly affecting hemispheric crus I, with atrophy of the folia and widening of the superior posterior and horizontal fissures. (g-h) Spinal mild volume reduction in dorsal and cervical segments. Patient D: T1 sagittal brain images (i: midsagittal; l: parasagittal), Coronal T2 brain image (m), T2 sagittal (n) and axial (o) cervical spinal images: (i) diffuse vermian atrophy associated with (l-m) cerebellar lobar atrophy with prevalent involvement of hemispheric crus I and widening of the superior posterior and horizontal fissures. (n-o) T2 hyperintensity in dorsal cervical spine

Table S1: Detailed description of patients' clinical characteristics					
**Symptoms**	**Patient A**	**Patient B**	**Patient C**	**Patient D**	**Patient E**
**Cough**	persistent cough that began at age 60	persistent cough that began at age 58	persistent cough that began at age 32	persistent cough that began at age 30	persistent cough that began at age 35
**Gait and postural symptoms**	The patient developed walking difficulties from the age of 68 with progressive ataxia in walking which caused progressive postural instability with episodes of falling. The patient currently walks independently without the need for aids. NE showed the presence of ataxic gait characterized by difficulty walking in a straight line, poor balance, and a widened base of support.	The patient developed walking difficulties from the age of 55 with progressive ataxia in walking which caused progressive postural instability. The patient currently walks independently without the need for aids. NE showed the presence of ataxic gait characterized by difficulty walking in a straight line, poor balance, and a widened base of support	The patient developed walking difficulties from the age of 62 with progressive ataxic gait. The patient currently walks independently without the need for aids. NE showed the presence of mild ataxic gait only characterized by a widened base of support	The patient developed walking difficulties from the age of 30 with progressive ataxia in walking which caused progressive postural instability. The patient currently walks independently without the need for aids. NE showed the presence of moderate ataxic gait characterized by difficulty walking in a straight line, poor balance, and a widened base of support	The patient developed walking difficulties from the age of 63 with progressive ataxia in walking. The patient currently walks independently without the need for aids. NE showed the presence of moderate ataxic gait characterized by difficulty walking in a straight line, poor balance, and a widened base of support
**Limb ataxia**	NE showed the presence of four limbs ataxia characterized by mild dysmetria on finger to nose and heel-to-shin tests	NE showed the presence of four limbs ataxia characterized by mild dysmetria on finger to nose and heel-to-shin tests	Not reported	NE showed the presence of limb ataxia characterized by mild dysmetria on finger to nose (left side) and heel-to-shin tests (bilateral)	NE showed the presence of four limbs ataxia characterized by mild dysmetria on finger to nose and heel-to-shin tests prevalent on the right side
**Sensory symptoms**	The patient has been complaining of numbness and tingling with a socking and glove distribution for some years associated with occasional cramps. NE showed the presence of diffuse areflexia and loss of sensation in the extremities	The patient has been complaining of numbness and tingling with a socking and glove distribution in the recent years. NE showed the presence of diffuse areflexia and loss of sensation in the extremities	The patient has been complaining of occasional numbness and tingling with a socking and glove distribution in the last two years. NE showed the presence of lower limbs areflexia and loss of sensation in the extremities	The patient has been complaining of occasional numbness and tingling with a socking and glove distribution since the age of 30. NE showed the presence of upper limbs hyporeflexia, lower limbs areflexia and loss of sensation in the extremities	The patient has been complaining of occasional numbness and tingling with a socking and glove distribution in the last years. NE showed the presence of lower limbs areflexia and loss of sensation in the extremities

Abbreviations: Neurological examination (NE).
